# Medical Physics 3.0, physics for every patient

**DOI:** 10.1002/acm2.12484

**Published:** 2018-10-19

**Authors:** Ehsan Samei, Michael D. Mills

**Affiliations:** ^1^ Duke University Durham NC USA; ^2^ Radiation Oncology University of Louisville Louisville KY USA

This issue, the JACMP presents a Guest Editorial authored by Ehsan Samei on the planned aspirations of the future of medical physics. I invite everyone to consider the opinion below and the reference publication on which it is based — Michael Mills.

Physics has done much in medicine. For one, it has been instrumental in the medical use of radiation, both ionizing and nonionizing. And in doing so, it has shaped the modern practice of diagnostic and therapeutic medicine. Over decades since the discovery of X‐rays, physics and physics principles have been steadily applied for continued advancement of technologies related to radiation medicine. Physics has also been present clinically and professionally in the *use* of radiation in medicine, where physicists play key roles in commissioning, inspecting, and optimizing the use of the technology. As the discipline has gradually specialized, its clinical presence has taken narrowly defined territories. This territorial definition, while professionally important, has created a constraint which is out of step with challenging needs that clinical medicine is facing, needs such as precision, personalization, and value‐based care that can be tackled by physics. As the profession is evolving, it is crucial to calibrate this evolution with the broader needs of medicine.

The realities noted above necessitate a new brand of medical physics with increased focus on the precision and personalization of patient care. The focus should include areas within and beyond radiation medicine. This new horizon calls for new skills for medical physicists to be able to practice with strong competence and confidence toward clinical impact. The AAPM initiated the Medical Physics 3.0 (MP3.0) as an effort to motivate this evolution of the profession. MP3.0 is thus defined as a new inspiration for physics serving every patient. The characteristics of this movement were recently clarified in an article in Medical Physics^1^ that I encourage every medical physicist to read and reflect upon.

You may ask, do we really need a new brand? Aren't we already impacting patient care? Why do we need Medical Physics 3.0? To these questions, I just offer a few answers in the limited space of an editorial.

1. Publicity: Many good things that we do already need to be better publicized. For example, a typical patient being benefited by our work should know what a medical physicist does, so do administrators who are resourcing healthcare. That is not the case today. We have an opportunity to improve this through MP3.0.
2. Inconsistency: Current practice, while great in many respects, is uneven across physics practices. Many places practice at the lowest common denominator. Case in point, we don't apply the same QC rigor across the clinical practice as we do for clinical trials. Is quality and safety for clinical practice any less important than those for clinical trials? Physicists have the ability and skill to make their contributions more *consistently* excellent everywhere. But to do so, we need a stronger peer expectation, an explicit regulatory mandate, better tools, and models of effective practice. We have an opportunity to improve upon these needs through MP3.0.
3. Relevance: We undertake many physics procedures. Some are not as relevant to medical care as others. For many we have a difficult task to relate physics results to clinical performance. In the era of value‐based care, the value of an undertaking that takes effort and expense needs to be justified. We have an opportunity to better delineate the relevance of physics in value‐based care through MP3.0.
4. Opportunity: Medical physics has definitely done much and continue to do so in enabling and advancing radiation medicine. But medical physics is not limited to radiation physics; physics is rather present in all aspects of health, healthcare, and associated technologies. Yet physicists are rarely present outsides of the departments of radiation oncology and radiology. We have an opportunity to improve this through MP3.0.

How is Medical Physics 3.0 bringing about what is needed? The ongoing activities of the initiative are orchestrated through Units, each of which is focused on fostering one of the seven “smart” aspirations of MP3.0. The term smart is used to invoke the character of intentionality, intelligence, and effectiveness in targeting the objectives of MP3.0. I encourage you to consider MP3.0 as an opportunity to make medical physics and medical physicists as relevant and as essential as they can be to healthcare. The primary reason to do so is not self‐serving, rather it is serving and advancing human health through intentionally comprehensive, integrated, and smart scholarship and practice.
